# ATP Exhibits Antimicrobial Action by Inhibiting Bacterial Utilization of Ferric Ions

**DOI:** 10.1038/srep08610

**Published:** 2015-02-25

**Authors:** Yutaka Tatano, Yuichi Kanehiro, Chiaki Sano, Toshiaki Shimizu, Haruaki Tomioka

**Affiliations:** 1 Department of Microbiology and Immunology, Shimane University School of Medicine, Izumo 693-8501, Japan; 2 Department of Nutrition Administration, Yasuda Women's University, Hiroshima 231-0153, Japan; 3 Faculty of Nursing, Yasuda Women's University, Hiroshima 231-0153, Japan

## Abstract

ATP up-regulates macrophage antimycobacterial activity in a P2X_7_-dependent manner, but little is known about whether ATP directly exhibits antimicrobial effects against intracellular mycobacteria. In this study, we found that ATP inhibited the growth of various bacteria, including *Staphylococcus*, *Pseudomonas*, and mycobacteria, without damaging bacterial surface structures. Using gene technology, we newly established an enterobactin-deficient (*entB*^−^) mutant from ATP-resistant *Klebsiella pneumoniae*, and found the recovery of ATP susceptibility in the enterobactin-deleted mutant. Therefore, ATP's antibacterial activity is attributable to its iron-chelating ability. Since ATP distributed in the cytosol of macrophages at high concentrations, ATP appears to augment macrophage's antimicrobial activity by directly attacking intracytosolic and intra-autophagosomal pathogens. Furthermore, ATP exhibited combined effects with some antimicrobials against methicillin-resistant *S. aureus* (MRSA) and *M. intracellulare*, suggesting its usefulness as an adjunctive drug in the chemotherapy of certain intractable infections.

ATP exhibits various biological activities, such as mitogenic stimulation and gene expression through ligation of P2 purinoceptors^1^,^2^. Macrophages (MΦs) possess both P2X_7_ and P2Y_2_ receptors^1^,^2^. ATP treatment of human MΦs potentiates their activity in killing mycobacterial organisms in a P2X_7_-dependent manner^3^. ATP-mediated killing of mycobacterial organisms within MΦ is mediated by phospholipase D, which is linked to leukocyte antimicrobial mechanisms dependent on the mobilization of intracellular Ca^2+^ and subsequent lysosomal fusion and acidification of mycobacteria-containing phagosomes^4^,^5^,^6^. ATP-mediated killing of intramacrophage mycobacteria is also attributable to MΦ apoptosis induced by P2X_7_-mediated signaling^7^. ATP signals transmitted through P2X_7_ receptors up-regulates MΦ antimycobacterial activity in a cytosolic phospholipase A_2_-dependent mechanism, and potentiated the therapeutic efficacy of chemotherapeutic agents against mycobacterial infection^8^. It is unknown which antimicrobial molecules act as effectors in intracellular mycobacterial killing by ATP-stimulated MΦs. One candidate is long chain free fatty acids, which are generated in the intraphagosomal milieu by the enzymatic action of cytosolic phospholipase A_2_^8^,^9^,^10^. MΦ treatment with high concentrations of ATP may cause the influx and accumulation of ATP in MΦ cytoplasm through large membrane pores generated in response to P2X_7_-mediated ATP signaling. Indeed, the long C-terminal tail of P2X_7_ allows its conformational change, causing the formation of a large-size pore, which permits transmembrane fluxes of small hydrophilic molecules, including ATP^11^. These situations encouraged us to examine the possibility that intracellularly condensed ATP molecules participate in the expression of MΦ antimycobacterial activity.

## Results

### ATP inhibits the growth of bacteria

**Figure 1:**
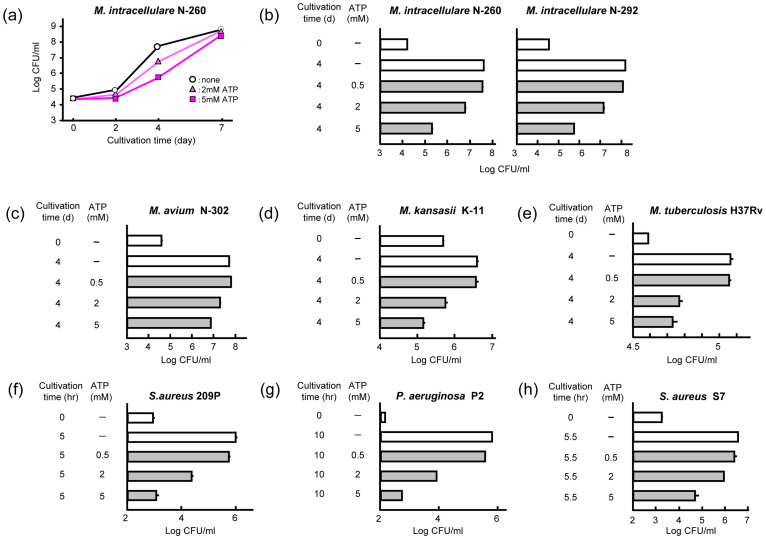
Antimicrobial activity of ATP against representative mycobacteria and common bacteria having ATP-susceptible phenotype. (a) Bacterial growth of *M. intracellulare* strain N-260 (serovar 16) in 7HSF medium in the presence or absence of ATP. (b–h) ATP-mediated growth inhibition of test bacteria. (b) *M. intracellulare* strains N-260 and N-292 (serovar 16). (c) *M. avium* strains N-302 (serovar 9). (d) *M. kansasii* strain K-11. (e) *M. tuberculosis* strain H37Rv. (f) *S. aureus* strain 209P. (g) *P. aeruginosa* strain P2. (h) *S. aureus* strain S7. Representative results obtained from at least two separate experiments are indicated.

First, we studied ATP's antimicrobial activity against extracellular *Mycobacterium intracellulare*. ATP (2 and 5 mM) markedly inhibited the growth of *M. intracellulare* during the first 4 days, but the delay of bacterial growth was recovered by day 7 (Fig. 1a). Serovar 16 strains were more susceptible to ATP than serovar 14 strains (Fig. 1b; Supplemental Fig. S1a). *M. avium* was moderately susceptible or resistant to ATP (Fig. 1c; Supplemental Fig. S1b). ATP susceptibilities of other mycobacterial species were as follows: *M. kansasii* was highly susceptible (Fig. 1d), *M. tuberculosis* was highly to moderately susceptible (Fig. 1e), and *M. fortuitum* was essentially resistant (Supplemental Fig. S1c). We then examined ATP susceptibility of common bacteria: *Staphylococcus aureus* was highly to moderately susceptible (Fig. 1f, h; Supplemental Fig. S1d), while *Listeria monocytogenes*, *Escherichia coli*, and *Klebsiella pneumoniae* were resistant to ATP (Supplemental Fig. S1e–g). ATP susceptibility of *Pseudomonas aeruginosa* markedly varied from strain to strain (Fig. 1g; Supplemental Fig. S1h). These findings indicate the species and strain dependency of common bacteria for their ATP susceptibility^12^. In separate experiments, we determined the effective concentrations of ATP which cause 50% inhibition of the growth of the two ATP-susceptible bacterial species with a serial dilution method, as follows: 0.60 mM for *P. aeruginosa* strain P2 and 0.77 mM for *M. intracellulare* strain N-260.

Next, we studied the mechanism of ATP's antimicrobial effects. Anti-*M. intracellulare* activity was shared by ATP, benzoylbenzoyl ATP (P2X_7_ agonist) and UTP (P2Y agonist), but not by AMP (P2Y agonist) (Supplemental Fig. S2a). In addition, oxidized ATP (P2X_7_ antagonist), and suramin, MIA and DIDS (P2X_7_ inhibitors) did not block ATP activity (Supplemental Fig. S2b, c). Therefore, ATP signaling through bacterial P2 purinoceptor-like molecules is not required for the expression of ATP's bacteriostatic action.

### Antimicrobial effect of ATP correlates with metal-ion chelating activity

**Figure 2:**
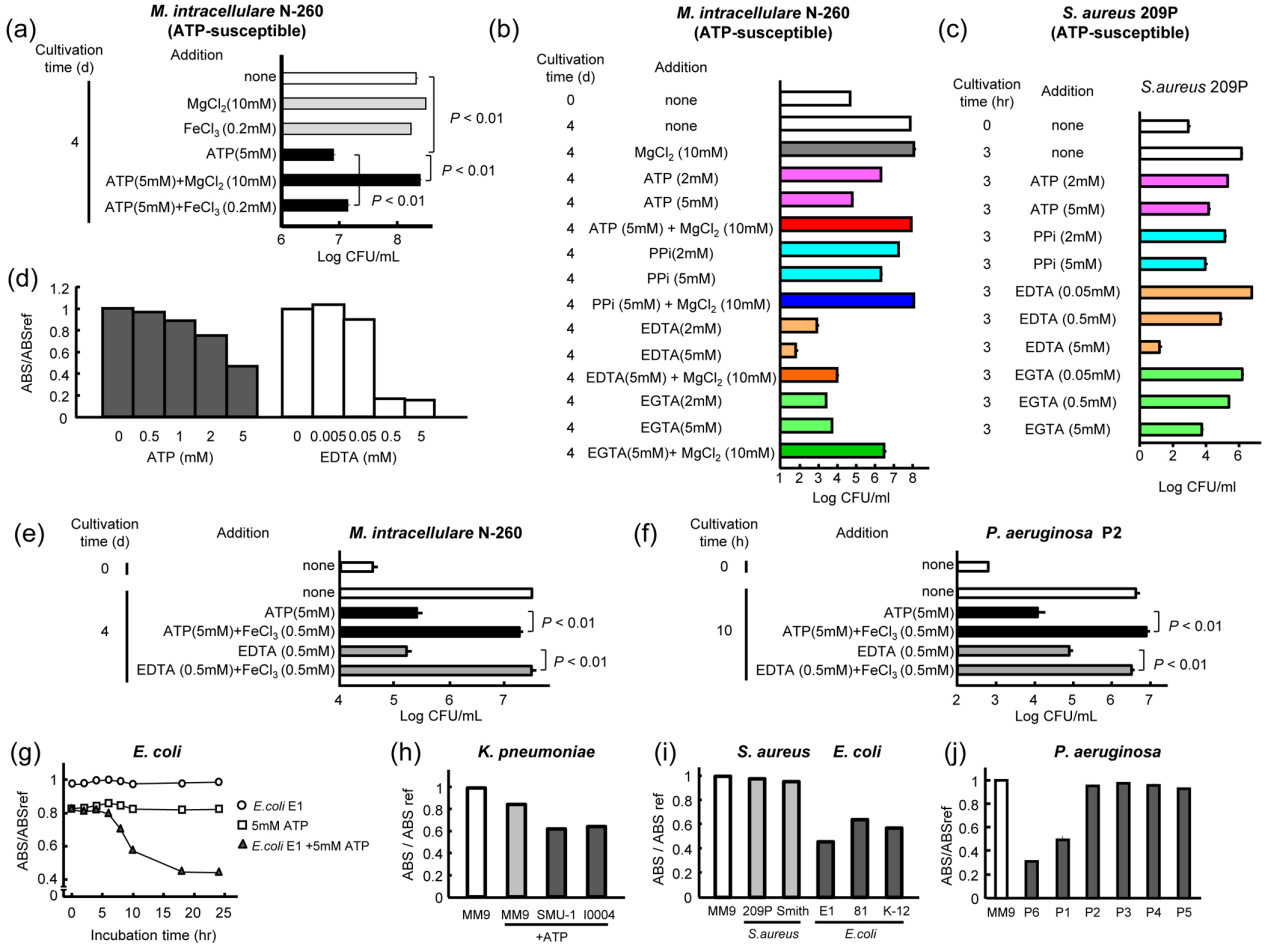
ATP's antimicrobial effect is attributable to its iron-chelating activity. (a) Blocking effects of MgCl_2_ and FeCl_3_ on ATP's anti-*M. intracellulare* antimicrobial activity. (b) Anti-*M. intracellulare* activities of ATP and various metal-chelating agents (pyrophosphate (PPi), EDTA, EGTA) and blocking effects of MgCl_2_. (c) Anti-*S. aureus* activities of ATP and various metal-chelating agents. (d) Ferric ion-chelating activities of ATP and EDTA measured by CAS assay. (e) Ferric ion-mediated reduction of antimicrobial effects of ATP and EDTA against *M. intracellulare*. (f) Ferric ion-mediated reduction of antimicrobial effects of ATP and EDTA against *P. aeruginosa*. (g) Siderophore production by ATP-resistant *E. coli* during the course of cultivation. (h) Siderophore production by two ATP-resistant *K. pneumoniae* strains during 48-h cultivation in the presence of ATP. (i) Siderophore production by ATP-susceptible *S. aureus* and ATP-resistant *E. coli* during 24-h cultivation in the presence of ATP (5 mM). (j) Siderophore production by ATP-susceptible and ATP-resistant strains of *P. aeruginosa* during 24-h cultivation.

ATP chelates metal ions, such as Mg^2+^ and Mn^2+^, causing modulation of various enzymatic reactions *in vivo*. Therefore, ATP may exhibit antimicrobial effects on certain microorganisms by depriving them of essential metal ions. This aspect was confirmed as follows. First, ATP's anti-*M. intracellulare* activity was significantly reduced by MgCl_2_ and FeCl_3_ (Fig. 2a). Second, all test metal chelators exhibited antimicrobial activity against *M. intracellulare* in the order of EDTA > EGTA > ATP > pyrophosphate (iron-chelator), and the excess free Mg^2+^ ion-occupying chelating ability of these agents partly blocked their antibacterial effects (Fig. 2b). Indeed, it has been demonstrated that EDTA exhibits strong antimicrobial activity against *P. aeruginosa*, and the antibacterial activity of certain antibiotics which inhibit bacterial protein synthesis was reported to be significantly potentiated when used in combination with EDTA, presumably due to the chelation of Ca^2+^ and Mg^2+^ ions^13^.

Antimicrobial effects of these chelators were markedly decreased when ATP low-susceptible *M. avium* was used as a target (Supplemental Fig. S3a). In common bacteria, a good correlation was also observed between their susceptibility to ATP and chelators (Fig. 2c; Supplemental Fig. S3b, c). Therefore, ATP's antimicrobial effect is attributable to its metal-ion chelating action.

Ferric ion is essential for the growth and survival of most living organisms^12^,^14^,^15^. During infection in host animals, bacterial organisms are obligated to compete with host iron-chelating substances, such as transferrin and lactoferrin, to acquire ferric ions. By using siderophores, bacterial high-affinity ferric chelators, bacteria maintain intracellular iron concentration at sufficient levels for their survival and growth in hosts^12^,^14^,^15^. As shown in Fig. 2d, ATP exhibited significant levels of ferric ion-chelating activity. In this context, we compared the profiles of the antibacterial actions of ATP with EDTA using *M. intracellulare* and *P. aeruginosa* as target organisms. ATP (5 mM) exhibited levels of growth-inhibiting activity against these bacteria comparable to those of EDTA (0.5 mM) (Fig. 2e, f). The combined use of FeCl_3_ (ferric ions) (0.5 mM) markedly reduced the antibacterial activity of ATP as well as EDTA, strongly suggesting that the antibacterial activities of ATP and EDTA are principally based on their ferric ion-chelating ability. Next, we found that ATP-resistant *E. coli* and *K. pneumoniae*, but not ATP-susceptible *S. aureus*, produced significant levels of siderophores when cultivated in the presence of 2 or 5 mM ATP (Fig. 2g–i). In *P. aeruginosa*, siderophore production was observed for only ATP-resistant strains (Fig. 2j). Therefore, the ATP-induced production of siderophores seems to be limited to ATP-resistant bacteria.

### Siderophore interferes with ATP's antimicrobial effects

**Figure 3:**
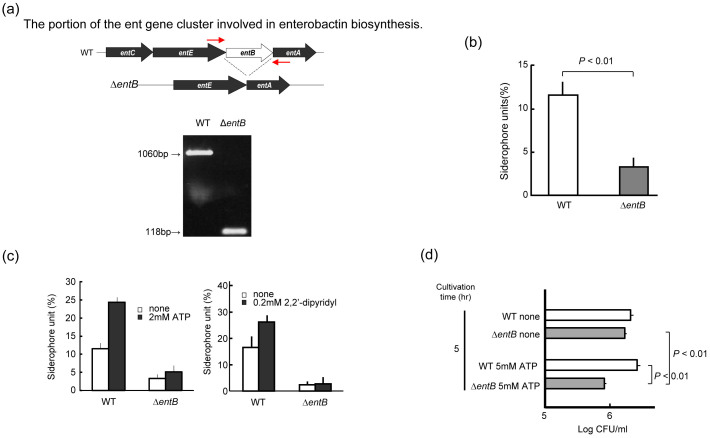
Enterobactin-deficiency restores ATP susceptibility of *K. pneumoniae*, which is intrinsically resistant to ATP's antimicrobial effects. (a) Scheme of the establishment of *entB*-KO *K. pneumoniae* mutant strain. (b) The *entB*-KO strain showed markedly decreased siderophore-producing ability. (c) Siderophore production by the *entB*-KO strain in the presence or absence of ATP and an iron-chelating agent dipyridyl. (d) Susceptibility of the *entB*-KO strain to ATP's antimicrobial effects.

Next, to confirm that the antibacterial activity of ATP is mediated by its iron-chelating effects, we established an enterobactin-deficient mutant strain of *K. pneumoniae* by deleting the *entB* gene encoding 2,3-dihydro-2,3-dihydrooxybenzoate synthase, a key enzyme for enterobactin biosynthesis (Fig. 3a)^16^. A PCR experiment confirmed that this strain has a complete deficit in the *entB* locus (Fig. 3a), resulting in a marked reduction in its siderophore-producing ability (Fig. 3b). While siderophore production by the parent ATP-resistant strain was augmented in the presence of ATP or another iron chelator 2,2′-dipyridyl, such a phenomenon was not observed in the *entB* gene-KO mutant strain. This suggests that the major siderophore, the production of which is up-regulated by ATP-mediated iron starvation, is enterobactin (Fig. 3c). Notably, ATP exhibited moderate levels of antimicrobial effects on the *entB*^−^ mutant strain, but not on the *entB*^+^ parent strain producing enterobactin (Fig. 3d). Therefore, it is concluded that the antimicrobial activity of ATP is mediated by its iron-chelating ability.

### ATP directly acts on intramacrophage bacteria in the cytosol

**Figure 4:**
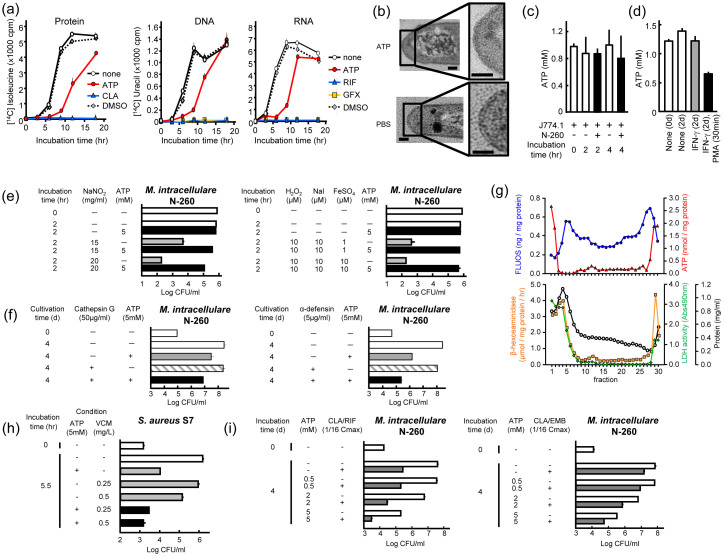
Roles of ATP as an intramacrophage antimicrobial effector and its usefulness in adjunctive chemotherapy for intractable bacterial infection. (a) Effects of ATP on macromolecule (protein, DNA, and RNA) synthesis by *S. aureus*. Test bacteria were cultured in the presence or absence of ATP (5 mM), clarithromycin (0.125 μg/ml), rifampin (0.125 μg/ml), or gatifloxacin (0.125 μg/ml). (b) Effects of ATP treatment on cell-surface structures of *M. intracellulare* assessed by electron microscopy. Test bacteria were incubated in PBS with or without the addition of 5 mM ATP at 37°C for 24 h, and subjected to electron microscopy. (c) Intracellular ATP concentration of MΦs with or without *M. intracellulare* infection. The cell lysate of test MΦs was prepared 2 and 4 h after bacterial infection (MOI = 10) and subjected to measurement of the ATP concentration by the luminol-luciferase assay using the ATP Bioluminescence assay kit CLS II (Roche). (d) Intracellular ATP concentration of MΦs after IFN-γ (200 units/mL)-priming with or without subsequent PMA (100 ng/mL)-triggering. (e) Anti-*M. intracellulare* activities of reactive nitrogen intermediates and H_2_O_2_-dependent halogenation reaction decreased in combination with ATP. (f) Anti-*M. intracellulare* activities of cathepsin G and α-defensin increased in combination with ATP. (g) Distribution profiles of ATP in phagosomes, lysosomes, and cytosol of *M. intracellulare*-infected J774.1 mouse MΦs. (h) Antimicrobial activity of vancomycin against *S. aureus* strain S7 belonging to MRSA increased in combination with ATP. (i) Anti-*M. intracellulare* activities of clarithromycin/rifampin and clarithromycin/ethambutol increased in combination with ATP. Abbreviations: VCM, vancomycin; CLA, clarithromycin; RIF, rifampin; EMB, ethambutol; GFX, gatifloxacin.

Next, we investigated the cellular mechanisms of ATP's antimicrobial action, as follows. First, ATP caused the early-phase inhibition of macromolecule synthesis (protein, RNA, and DNA) by *S. aureus*, but the inhibition thereafter diminished, presumably due to genetic adaptation to iron starvation (Fig. 4a)^17^. Second, ATP exhibited no bactericidal effect on *M. intracellulare* (Supplemental Fig. S4a) and caused no damage to the bacterial surface structure, including cell membranes, in terms of the release of intracellular proteins (Supplemental Fig. S4b). This aspect is supported by electron microscopy of the surface structure of ATP-treated *M. intracellulare*, indicating no obvious damage to the cell wall and cytoplasmic membrane (Fig. 4b). Third, intracellular concentrations of ATP in various cells including MΦs are reported to be 1 to 8 mM^18^,^19^. Therefore, MΦs are capable of acquiring sufficient intracellular ATP concentration to exhibit antimicrobial effects.

Based on this, we carried out some experiments to confirm the relationship between MΦ antibacterial activity and the intramacrophage ATP concentration, and obtained the following findings. Firstly, J774.1 MΦs were measured for changes in the intracellular ATP concentration during the course of *M. intracellulare* infection. Interestingly, the intracellular ATP concentration was maintained at around 1 mM for up to 4 h after bacterial infection, showing that the intracellular paucity of ATP did not occur in MΦs because of bacterial invasion (Fig. 4c). Importantly, this finding indicates that, in MΦs engulfing bacteria, sufficient intracellular ATP concentrations for the efficacious expression of ATP antibacterial activity are maintained during the course of infection. In addition, pretreatment of MΦs with a representative MΦ-activating cytokine, IFN-γ, with or without subsequent triggering with phorbol myristate acetate (PMA), did not cause a significant increase in the intracellular ATP concentration (Fig. 4d). This finding suggests that the ATP's antimicrobial action against intramacrophage bacteria is not potentiated in connection with MΦ activation elicited by IFN-γ-priming and PMA-triggering. It thus appears that intracytosolic ATP only partially contributes to the manifestation of the antibacterial activity of MΦ as a basic intramacrophage antimicrobial system. Notably, PMA-triggering caused a marked decrease in the intramacrophage ATP concentration, suggesting the rapid consumption of intracytosolic ATP, presumably due to the PMA-induced potentiation of intracellular metabolic pathways related to inflammatory reactions elicited by PMA-mediated signaling.

Since MΦ apoptotic events lead to the elevation of cytosolic ATP concentrations, ATP may also contribute to the apoptosis-mediated induction of MΦ antimycobacterial activity^19^. Thus, ATP is expected to participate in the expression of MΦ's antimycobacterial function. Interestingly, ATP exhibited antagonistic effects on the bactericidal activity of reactive nitrogen intermediates and reactive oxygen intermediates, the major intraphagosomal antimicrobial effector molecules of MΦs (Fig. 4e), while it exhibited additive effects in combination with cationic antimicrobial peptides (Fig. 4f). Next, we examined whether or not ATP is co-localized in MΦ phagosomes engulfing bacteria. ATP was recovered from top and bottom fractions corresponding to the cytosol and possibly also in the lysosomal vesicles (Fig. 4g). Although *M. intracellulare*-engulfing phagosomes were also sedimented at bottom position, its peak fraction (Fr. 28) did not correspond to the position of ATP sedimentation. Taken together, ATP seems not to actively participate in intraphagosomal killing of *M. intracellulare*, but plays important roles in the growth inhibition of the organisms residing in the cytosol of MΦs.

### ATP enhances the activity of antimicrobial drugs for clinical treatment of intractable infections

Iron acquisition in the host milieu is critical for the expression of virulence by *Vibrio vulnificus*, *M. tuberculosis*, and fungi. Thus, iron overload in hosts occasionally causes the progression and deterioration of infection due to these pathogens^12^,^14^,^15^. Hence, trials of iron-chelating therapy have been carried out^12^,^20^. Here, we examined the profile of ATP's antimicrobial activity from the viewpoint of its application to the treatment of bacterial infections. Notably, ATP augmented the antimicrobial activity of vancomycin against MRSA (Fig. 4h). ATP also potentiated the antimicrobial activity of clarithromycin/rifampin and clarithromycin/ethambutol regimens against *Mycobacterium avium* complex (MAC) (Fig. 4i). Thus, it is promising to use ATP as an adjunctive agent in chemotherapy for intractable MRSA and MAC infections. Indeed, ATP is widely distributed within the animal body, is essentially non-toxic, and exhibits no severe side effects on the human body.

## Discussion

In this study, ATP was found to exhibit growth inhibitory activity against some kinds of bacteria, including *S. aureus*, *P. aeruginosa*, and slowly growing mycobacteria. In this context, it has been reported that mammalian siderophore, 2,5-dihydroxy benzoic acid, promotes the growth of *E. coli* under the Fe^3+^-depleted condition and consequently causes the reduction of host resistance to *E. coli* infection^21^. Interestingly, ATP having iron-chelating activity behaved in a manner strikingly opposite to the mammalian siderophore. Notably, ATP does not facilitate but interferes with bacterial iron uptake.

Important findings concerning the biological mechanisms of ATP's antimicrobial effects are as follows. ATP exhibited ferric ion-chelating activity and ATP's antimicrobial activity was mimicked by a ferric ion-chelating agent pyrophosphate and metal ion chelators, EDTA and EGTA. Indeed, the occupation of ferric ion-binding activity of ATP with MgCl_2_ and FeCl_3_ blocked ATP's antimicrobial action. Chrome Azurol S (CAS) assay indicated that ATP-resistant bacterial organisms, but not ATP-susceptible organisms, produced ferric ion chelators. Importantly, ATP-resistant *K. pneumoniae* strain acquired ATP susceptibility by losing its ability to produce enterobactin due to deletion of the *entB* gene. These findings support the very novel view that ATP exhibits antimicrobial effects by depriving ferric ions from target bacteria through its iron-chelating effects.

With special reference to the biological significance of ATP's antimicrobial effects, it is of interest to clarify whether ATP plays significant roles in the antimicrobial mechanisms of host MΦs. Among MΦ organelles, ATP was specifically localized in the cytosol and possibly also in the lysosomal vesicles, as previously reported^22^,^23^, but not in *M. intracellulare*-engulfing phagosomes (Fig. 4g). Notably, the anti-*M. intracellulare* activity of reactive nitrogen and oxygen intermediates, the most important antimicrobial effectors of MΦs, was completely blocked by ATP (Fig. 4e), although ATP potentiated the anti-*M. intracellulare* activity of the cationic antimicrobial peptide cathepsin G, a lysosomal antimicrobial serine protease (Fig. 4f). Taken together, we can exclude the possibility that ATP plays central roles in intraphagosomal inhibition and the killing of infected mycobacteria inside MΦs. It is possible that ATP plays important roles in the inhibition of bacterial growth in the cytosol of MΦs. In this context, it is generally considered that the intramacrophage survival and growth of virulent mycobacteria occur mainly due to the inhibition of phagosomal maturation and phagosome-lysosome fusion by mycobacterial virulence factors, including lipoarabinomannan, protein kinase G, and protein phosphatase A^24^,^25^. Notably, van der Wel *et al*. reported the opposite finding that phagosome-lysosome fusion rapidly occurs even in MΦs engulfing virulent mycobacteria including *M. tuberculosis*^26^. They found that live *M. tuberculosis* translocates from phagolysosomes into cytosol, and cytosolic entry is dependent on bacterial secretion of ESAT-6 and CFP-10 proteins^26^. Indeed, after phagocytic internalization into host MΦs, *M. tuberculosis* and MAC escape from phagosomes into cytosol with the aid of *tlyA* gene-encoded cytolysin^26^,^27^. Thus, bacterial evasion of MΦ's intraphagosomal killing system is an important strategy of virulent mycobacteria to survive inside host MΦs. The present findings suggest that ATP acts as an inhibitor of the intracytoplasmic growth of bacteria, which evade MΦ phagosomes after infecting host MΦs via phagocytosis.

In this context, autophagosome-mediated bacterial inhibition may be important for the elimination of intracytosolic mycobacteria, which evade intraphagosomal killing. Indeed, autophagy plays an important role in host immunological defenses against pathogenic mycobacteria^28^. Therefore, there is a possibility that autophagic events in infected MΦs may be connected with ATP-mediated upregulation of the antimycobacterial function of host MΦs. IFN-γ-induced autophagy, causing the maturation of mycobacteria-containing autophagosomes to autophagolysosomes, is effective in the elimination of mycobacteria that have escaped from phagosomes into cytosol^29^. Furthermore, ATP induces autophagy in MΦs and causes rapid bacterial killing within autophagosomes^30^. Therefore, it is likely that cytosolic ATP is accumulated in autophagosomes together with mycobacterial organisms and exhibits antimicrobial action against bacteria residing within autophagolysosomes. This view should be confirmed by systematic experiments on the autophagosome-mediated killing of intracytosolic bacteria, and further studies are currently underway.

## Methods

### Organisms

Five strains each of *M. avium*, strain N-254 (serovar 9), N-302 (serovar 9), N-339 (serovar 8), N-444 (serovar 8) and N-445 (serovar 1), and *M. intracellulare*, strain N-244 (serovar 14), N-260 (serovar 16), N-285 (serovar 16), N-291 (serovar 14) and N-292 (serovar 16) were used. These MAC strains were isolated from sputum specimens of patients with pulmonary MAC infection in Japan and identified by a DNA probe test. In addition, the following microorganisms were used: three strains of *M. tuberculosis* (strain H37Rv, H37Ra and Kurono), two strains of *M. kansasii* (strain K-5 and K-11), *M. fortuitum* (strain F-1, F-19, F-20), and common bacteria including *S. aureus* (strain 209P, Smith, S2, S3, S4, S5, S6, S7), *L. monocytogenes* strain EGD, and *E. coli* (strain K-12, 81, E1, E2, E3, E4, E5, E6), *K. pneumoniae* (strain I0004, I0008, I0029, I0036, I0058, SMU-1), and *P. aeruginosa* (strain P1, P2, P3, P4, P5, P6). Test mycobacteria and common bacteria were grown in Middlebrook 7H9 medium and Tryptic soy broth, respectively.

### Special agents

The following agents were used: ATP (Sigma Aldrich Co., St. Louis, MO; MP Biomedicals, Solon, OH, USA, Calbiochem Co., La Jolla, CA, and Roche Diagnostic Co., Indianapolis, IN), ADP (Sigma), AMP (Sigma), adenosine (Sigma), benzoylbenzoyl ATP (Sigma), oxidized ATP (Sigma), suramin (Wako, Tokyo, Japan), MIA (methyl isobutyl amiloride, Wako), DIDS (4,4′- Diisothiocyanatostilbene-2,2′-disulfonic acid, Sigma), EDTA (Dojindo, Tokyo, Japan), EGTA (Dojindo), dipyridyl (Sigma), CAS (Chrome Azurol S, Sigma), *E. coli* S17-1 and pK18mobSacB (kindly provided by Dr. F. Taguchi, Okayama University), Instagene matrix (Bio-Rad, Hercules, CA), KOD-Plus (Toyobo, Osaka, Japan), Wizard SV Gel and PCR cleanup system (Promeg, Madison, WI), Ligation High (Toyobo), Protein Assay Rapid Kit (Wako), [^14^C] isoleucine (Moravek Biochemicals, Inc, Brea, CA), [^14^C]uracil (Moravek Biochemicals, Inc.), α-defensin-1 (Peptide institute, Inc, Osaka, Japan.), cathepsin G (Sigma), vancomycin (Wako), clarithromycin (Taisho-Toyama Pharmaceutical Co., Tokyo), rifampin (Daiichi Sankyo Co., Tokyo), and ethambutol (Sigma), gatifloxacin (Wako), FLUOS (Sigma), LB medium (Invitrogen, San Diego, CA), M9 medium (prepared by our laboratory), Heart infusion agar (Eiken Chemical Co., Tokyo, Japan), Middlebrook 7H9 medium (Becton Dickinson, Cockeysville, MD), and Middlebrook 7H11 medium (Becton Dickinson).

### Mice

Twelve-week-old male BALB/c mice purchased from Japan Clea Co., Osaka, Japan, were used. Animal care and experimental procedures were approved by the Animal Research Committee of Shimane University and conducted according to the Regulations for Animal Experimentation at Shimane University.

### Antimicrobial activity of test agent against extracellular organisms

The activities of test agents against extracellular mycobacteria were measured as follows. Test organisms (10^5^ CFUs) were suspended in 0.5 ml 7HSF medium, a broth medium with the same composition as 7H11 agar without malachite green, containing test agents, and cultivated at 37°C in a CO_2_ incubator (5% CO_2_-95% humidified air). After 4-d cultivation, the incubation mixture was centrifuged at 1,000 × *g* for 10 min, and the recovered micro-organisms were then washed with distilled water by centrifugation. The resulting bacterial pellet was suspended in 0.5 ml distilled water and the CFUs of recovered organisms were counted on the 7H11 agar plate. Next, in cases of common bacteria, test microorganisms were cultivated in Tryptic soy broth containing the test agents at 37°C for 3 to 10 h. The CFUs in the resultant culture were counted on heart infusion agar plates.

### Measurement of bactericidal activities of MΦ's antimicrobial effectors

*M. intracellulare* strain N-260 was incubated in 0.1 M sodium acetate buffer (pH 5.5) with or without the addition of prescribed concentrations of (1) cathepsin G, α-defensin, NaNO_2_, or H_2_O_2_ + NaI + FeSO_4_ at the cell density of 1 × 10^6^ CFU/ml at 37°C. After individual treatment of test microorganisms for 2 h or 4 d, residual bacterial CFUs were counted on 7H11 agar.

### Establishment of enterobactin-deficient mutant strain of *K. pneumoniae*

An enterobactin-deficient mutant strain of *K. pneumoniae* was established by deleting the *entB* gene (Fig. 3a). Briefly, plasmid DNA was constructed by conjugating a negative-selection suicide vector containing the *sacB* gene and the *K. pneumoniae* enterobactin locus, from which the *ent*B gene was deleted (Fig. 3a: upper illustration). The plasmid was transfected into *E. coli* strain S-17-1, and the resultant transformant was co-cultivated with *K. pneumoniae* strain SMU-1 to allow recombination between the transfected enterobactin locus and the same DNA region of the parent *K. pneumoniae* strain, providing an *entB* deletion mutant. PCR experiment confirmed that this strain has a complete deficit in the *entB* locus (Fig. 3a). The detailed experimental procedures are as follows. The whole genome DNA of *K. pneumoniae* strain SMU-1 (wild-type strain) was prepared using Instagene matrix. To generate a deletion mutant of the *entB* gene, upstream (1518 bp) and downstream (1133 bp) regions of its ORF were amplified using two PCR primer sets: (1) *entB*-F1-Sal (forward primer), 5′-cagctggtcgacaaccttgcctg-3′, and *entB*-R1-Nh (reverse primer), 5′-ttcgccgggctagcgctggcttc-3′, and (2) *entB*-F2-Nh (forward primer), 5′-aggctagccgtttgccaccgaaac-3′, and *entB*-R2-RI (reverse primer), 5′-agcaggggaattcccggatggcgg-3′. The resultant DNA amplicons were subjected to enzymatic digestion with Sal I and Nhe I endonucleases and with Nhe I and EcoR I endonucleases, and the obtained DNA fragments were then integrated into a pK18mobSacB plasmid DNA between its Sal I and Eco RI restriction sites. After electroporation-based incorporation of the resultant plasmid DNA into *E. coli* strain S17-1, the plasmid was further transferred to *K. pneumoniae* strain SMU-1 via bacterial conjugation, allowing homologous recombination between the plasmid DNA and the corresponding genetic region of the host genome DNA. Then, bacteria having incorporated plasmids were deleted by performing cultivation on an LB agar plate containing 10% sucrose. The deletion of the *entB* gene in the recombinant *K. pneumoniae* was confirmed by genomic PCR testing using a primer set: *entB*-F3 (forward primer), 5′-ccgaaggcaagctgggctgaaggag-3′, and *entB*-R3 (reverse primer), 5′-gcatcggcgacgtccagcgtttcgg-3′.

### Measurement of bacterial siderophore production by the CAS assay

Bacterial siderophore production was measured by the CAS assay, as follows. Test bacteria were cultured in LB medium with or without the addition of prescribed concentrations of ATP or dipyridyl at 37°C for 1 h. The resultant bacteria were then collected by centrifugation, re-suspended in modified M9 minimal medium, and cultivated at 37°C for 48 h. The resultant culture fluid was mixed with an equal volume of CAS reagent, and the OD_620_ of the resultant mixture was measured after 15-min incubation at room temperature. Siderophore production was represented by the decrease in the OD_620_ value from that of the control incubation mixture without the addition of bacteria.

### Measurement of macromolecule biosynthesis by *S. aureus*

*S. aureus* strain 209P was cultured in the presence or absence of ATP at a cell density of 5 × 10^5^ CFU/ml in 0.2 ml Tryptic soy broth containing [^14^C] isoleucine (1 μCi/ml) or [^14^C] uracil (0.1 μCi/ml) for the measurement of protein and DNA/RNA synthesis, respectively, in a microculture well for up to 20 h. For the measurement of protein and whole nucleic acid (DNA plus RNA) synthesis, the bacterial culture was added to 1% bovine serum albumin, the bacterial culture was spotted onto paper filter (Whatmann, Maldstone, England). The paper filter was thereafter rinsed twice with 10% trichloroacetic acid solution and with ethanol once, dried at 60°C, immersed in toluene-based scintillant, and counted for radioactivity using Tri-Carb 2100TR (Perkin Elmer, Inc., Waltham, MA). For the measurement of whole DNA synthesis, the bacterial culture was pretreated with 1 M KOH for 24 h and subjected to the same procedures as for protein and whole nucleic acid measurement, as described above.

### Measurement of ATP concentrations in MΦ's organelles and cytosol

J774.1 mouse MΦs (4 × 10^7^), which had been pretreated with interferon-γ (50 U/ml) for 24 h, were cultivated at the cell density of 8 × 10^6^ cells/dish in 10% fetal bovine serum-RPMI 1640 medium in the presence of 4 × 10^8^ CFU/dish FLUOS-labeled *M. intracellulare* strain N-260 (MOI = 50) at 37°C for 2 h. Infected MΦs were harvested by centrifugation, suspended in 1 ml of 0.25 M sucrose buffer containing protease inhibitors (PMSF, leupeptin, pepstatin A, and EDTA), and homogenized using a Dounce homogenizer (Wheaton Science Products, Millville, NJ). After 5-min centrifugation at 200 × *g*, the resultant supernatant was subjected to Percoll-gradient ultracentrifugation. Briefly, the supernatant was mixed with 9 volumes of 18% Percoll and centrifuged at 36,000 × *g* for 1 h. After centrifugation, the Percoll gradient formed in the centrifuge tube was fractionated into 30 fractions, and each fraction was measured for FLUOS fluorescence intensity (excitation at 488 nm and emission at 535 nm) and its ATP concentration was determined by the usual luciferase assay. In addition, β-hexosaminidase (lysosomes) and lactate dehydrogenase (cytosol) were measured in the gradient fractions by the usual methods.

### Effects of ATP on cell-surface structures of mycobacteria

ATP-mediated impairment of the bacterial cell membrane was measured in terms of the release of cellular proteins, as follows. *M. intracellulare* strain N-260 was incubated in phosphate-buffered saline (PBS) with or without the addition of 5 mM ATP at 37°C for up to 96 h. At intervals, the bacterial culture was harvested, centrifuged at 5,000 × *g* for 10 min, and the resultant supernatant was measured for the concentration of cellular proteins released from bacterial cell bodies by the Bradford assay. Furthermore, bacterial surface structures were observed directly using transmission electron microscopy. According to the usual method, test bacterial cells were treated with glutaraldehyde-based fixative solution, embedded in epoxy resin, and polymerized at 60°C. For ultrastructural studies, thin sections were stained with uranyl acid and lead citrate prior to examination using a transmission electron microscope at a JEM-1200EX (JEOL, Tokyo, Japan).

### Statistical analysis

Statistical analysis was performed by Bonferroni's multiple *t*-test using StatView software (HULINKS, Inc., Tokyo, Japan).

## Author Contributions

H.T. supervised this study, designed the experiments, and wrote the manuscript. Y.T. designed and performed the experiments, wrote some parts of the manuscript and prepared all the figures. H.T. and Y.T. analyzed the data. Y.K. partly designed and performed the experiments. C.S. and T.S. partly performed the experiments.

## Supplementary Material

Supplementary Information Supplementary informations
