# Supplementary material for: ATP Exhibits Antimicrobial Action by Inhibiting Bacterial Utilization of Ferric Ions
Source: Sci Rep. 2015 Feb 25;5:8610. doi: 10.1038/srep08610 (PMC4339799; doi:10.1038/srep08610)
Supplement: Supplementary Information — s [file srep08610-s1.doc]

**ATP Exhibits Antimicrobial Action by Inhibiting Bacterial Utilization of Ferric Ions**

**Yutaka Tatano, Yuichi Kanehiro, Chiaki Sano, Toshiaki Shimizu, and Haruaki Tomioka**

**Supplementary informations**

**Strain dependence of ATP susceptibility of tested bacterial species**

Some bacterial species showed strain dependence in terms of their susceptibility to ATP’s antimicrobial activity, as follows (Fig. 1; Supplemental Fig. S1 and unpublished observations). In *M. intracellulare*, two strains (N-260: serovar 16 and N-292: serovar 16) were highly susceptible to ATP, but the other three strains (N-244: serovar 14, N-285: serovar 16, and N-291: serovar 14) were resistant. In *M. avium*, one strain (N-302: serovar 9) was moderately susceptible, but the other four strains (N-254: serovar 9, N-339: serovar 8, N-444: serovar 8, and N-445: serovar 1) were resistant. In *M. kansasii,* one strain (K-11) was highly susceptible and another strain (K-5) was highly to moderately susceptible. In *M. tuberculosis*, all three strains (H37Rv, H37Ra, and Kurono) were moderately susceptible. In *M. fortuitum*, all three strains (F-1, F-19, and F-20) were resistant. In *S. aureus*, five strains (209P, S2, S3, S4, and S7) were highly susceptible, one strain (S5) was moderately susceptible, and the other two strains (Smith and S6) were resistant. In *L. monocytogenes*, one strain (EGD) was resistant. In *E. coli*, all seven strains (K-12, 81, E1, E2, E3, E4, E5, and E6) were resistant. In *K. pneumoniae*, all six strains (I0004, I0008, I0029, I0036, I0058, and SMU-1) were resistant. In *P. aeruginosa*, four strains (P2, P3, P4, and P5) were highly susceptible, but the remaining two strains (P1 and P6) were resistant.

**Evidence that ATP’s antimicrobial activity is not dependent on bacterial P2 purinoceptor-like molecules**

Several ATP preparations purchased from different companies (MP Biomedical, lot No. 6552H and 9082H; Sigma, lot No. 026K1561; Calbiochem, lot No. B63308; Roche, lot No. 93398121) exhibited similar levels of growth inhibitory activity against *M. intracellulare*, indicating that the ATP molecule itself is responsible for the observed antimicrobial activity (unpublished observation). Anti-*M. intracellulare* antimicrobial activity of various purines was assessed as follows. ADP and adenosine exhibited weak, while AMP exerted no antimicrobial action (Supplemental Fig. S2a). A potent P2X_7_ agonist, 2’(3’)-O-(4-benzoylbenzoyl) ATP (BzATP), exhibited markedly greater activity than ATP, suggesting that ATP’s action may be connected with bacterial signaling pathways mediated by P2X_7_-like molecules (Supplemental Fig. S2a). However, this possibility was excluded because a strong P2X_7_ antagonist, oxidized ATP (oATP), failed to block the expression of ATP’s antimicrobial activity, while exhibiting anti-*M. intracellulare* activity and additive effects with ATP (Supplemental Fig. S2b). Moreover, other P2X_7_ inhibitors, including suramin, MIA, and DIDS, also failed to block ATP’s antimicrobial activity (Supplemental Fig. S2c). In this context, AMP, a P2Y receptor agonist, showed no antimicrobial activity, while another P2Y agonist, UTP, exerted strong activity (Supplemental Fig. S2a). Therefore, ATP’s antimicrobial activity seems not to be mediated by P2 receptor-like molecules.

**Figure S1. Antimicrobial activity of ATP against representative mycobacteria and common bacteria having ATP-resistant phenotype. (a-h)** ATP-mediated growth inhibition of test bacteria. **(a)** *M. intracellulare* strains N-291 (serovar 14). **(b)** *M. avium* strain N-339 (serovar 8). **(c)** *M. fortuitum* strain F-1. **(d)** *S. aureus* strain Smith. **(e)** *L. monocytogenes* strain EGD. **(f)** *E. coli* strain 81, **(g)** *K. pneumoniae* strain SMU-1, **(h)** *P. aeruginosa* strain P6. Representative results obtained from at least two separate experiments are indicated.

**Figure S2. ATP’s antimicrobial effect is not mediated by bacterial P2 purinoceptor-like components. (a)** Anti-*M. intracellulare* activities of adenine nucleotides, adenosine, and UTP. **(b)** Effects of P2X_7_ an antagonist oATP on ATP’s anti-*M. intracellulare* activity. **(c)** Effects of P2X_7_ inhibitors (suramin, MIA, DIDS) on ATP’s anti-*M. intracellulare* activity.

**Figure S3. ATP-resistant bacterial strains are also resistant to metal-chelating agents. (a-c)** Antimicrobial effects of ATP and various metal-chelating agents (pyrophosphate, EDTA, EGTA) against *M. avium* strain N-444 **(a)**, *L. monocytogenes* strain EGD **(b)**, and *E. coli* strain 81 **(c)**.

**Figure S4. ATP does not affect bacterial viability and the intactness of cell membranes of *M. intracellulare*. (a)** Changes in bacterial viability during 96-h treatment with 5 mM ATP. **(b)** Release of cytosolic proteins from *M. intracellulare* due to ATP treatment.
